# Knowledge, attitudes and practices concerning epilepsy among nurses and midwives working in primary health care settings in Ouagadougou of Burkina Faso

**DOI:** 10.1186/s42494-023-00123-6

**Published:** 2023-05-15

**Authors:** Alfred Anselme Dabilgou, Alassane Dravé, Julie Marie Adeline Wendlamita Kyelem, Fatimata Kinda, Christian Napon, Athanase Millogo, Jean Kaboré

**Affiliations:** 1 Department of Neurology, University Hospital Yalgado Ouedraogo, Box 7022, Ouagadougou 03, Burkina Faso; 2 Department of Neurology, Regional University Hospital of Ouahigouya, 03 BP 7009 Ouagadougou 03, Burkina Faso; 3 Department of Neurology, University Hospital of Bogodogo, 14 Box 371, Ouagadougou 14, Burkina Faso; 4 Department of Neurology, Souro Sanou University Teaching Hospital, 01 Box 676, Bobo Dioulasso, Burkina Faso

**Keywords:** Epilepsy, Knowledge, Attitude, Practice, Paramedics, Ouagadougou, Burkina

## Abstract

**Background:**

In developing countries, there is a lack of epilepsy knowledge among health workers. The objective of this study was to assess the knowledge, attitude and practice concerning epilepsy among nurses and midwives working in primary health care settings in Ouagadougou.

**Methods:**

We carried out a cross-sectional study in the health districts of Ouagadougou from August 1st to September 15th, 2017. All nurses and midwives working in three health districts were included in this study.

**Results:**

A total of 213 participants with a mean age of 39.5 years were included in the survey; 79.81% of them had a general certification in secondary education and 62% had a professional experience of more than 10 years. About 99% of the participants had not received training on epilepsy-related care during the last six months. In addition, 74.5% of the participants had a good knowledge on epilepsy and 65% had a good practice toward epilepsy. The level of knowledge was associated with the workplace, years of training, and the professional experience. The level of knowledge about epilepsy was also associated with the level of education, while there was a significant link between professional status and nurses' level of practice in the management of seizures.

**Conclusions:**

Efforts must be made to provide continuing education for nurses in order to improve their knowledge on epilepsy.

## Background

There are over 70 million people estimated to be affected with epilepsy worldwide, with approximately 80% being living in developing countries where epilepsy is under-diagnosed and often untreated [1, 2]. In Sub Saharan Africa, almost 60% of people with epilepsy fail to receive medication and only about 33% of those who receive medications are appropriately managed [3]. In developing countries epilepsy is frequently managed by general physicians and nurses [4, 5]. Nurses and non-medical health workers are often the only health staff available to diagnose people with epilepsy [6]. Several studies have demonstrated an overall lack of epilepsy knowledge among physicians and medical students [5, 7].

In Burkina Faso, epilepsy is often neglected due to the insufficiency of expertise in neurology among physicians in primary health care [8]. There are few studies assessing the knowledge on epilepsy among health workers, including head nurses, members of the district health management team and district health unit supervisors [9, 10]. Studies showed weak knowledge on epilepsy. In addition, no studies have assessed the knowledge, attitude and practices of clinical nurses and midwives working at the forefront to see patients with first seizures in health districts. The aim of this study was to evaluate their knowledge and practices in order to improve care for and to reduce stigmatization of patients with epilepsy.

## Methods

### Study setting

This study was conducted in Ouagadougou, the capital city of Burkina Faso. Burkina Faso is a French-speaking country located in West Africa. It covers an area of ​​274,200 km^2^. In 2017, its population was estimated to be 16,632,147 [8]. It is bounded by Mali to the north and west, Togo and Ghana to the south, Benin to the southeast, Ivory Coast to the southwest, and Niger to the east. Based on administrative organization, Burkina Faso is divided into 13 regions, which are then divided into 45 provinces and subdivided into ~ 351 communes and 8,000 villages. The health supply is insufficient in Burkina Faso, with ratios of one doctor per 14,404 inhabitants, one state nurse per 3619 inhabitants and one midwife per 5874 inhabitants [8].

### Health system in Burkina Faso

The healthcare services in Burkina Faso are organized on three levels. The first level is made up of health and social promotion centers (CSPS), medical centers and district hospitals. The CSPS are the basic and most numerous healthcare units, generally including a dispensary, a maternity unit and a depot for essential generic drugs. CSPS mainly provide services of curative nursing consultations, vaccinations, consultations of healthy infants, deliveries, prenatal consultations and familial planning. Medical centers, headed by doctors, offer CSPS services, medical services, and specialized paramedical services, and have diagnostic supporting structures such as laboratories and medical imaging facilities. The district referral hospitals (CMAs) are the referral center for first-level health facilities at the health district level. They provide support for cases referred by first-level health facilities, support for medical, surgical and gyneco-obstetrical emergencies. The main activities are patient hospitalization, laboratory investigations and medical imaging. At the administrative level, patients are referred to and contra-referred to at the tertiary level. They are responsible for referrals and cross-referrals at the tertiary level. They also collect, process and analyze health data. The second level consists of regional hospitals to which the CMAs must refer their patients. As such, the regional referral hospitals (CHR) ensure prevention of health risks, and offer diagnosis, treatment and monitoring of diseased or injured patients as well as pregnant women. The third level is made up of university hospital centers. In 2017, the city of Ouagadougou had 109 public health facilities, covering five health districts (Baskuy, Bogodogo, Boulmiougou, Nongr-Massom and Sig-Nonghin). The five districts had five CMAs, 83 CSPS, nine isolated dispensaries, one isolated maternity unit and nine garrison infirmaries.

### Organization of nursing schools

Nurses in Burkina Faso are mainly trained for supportive roles. Their training is assured by National School of Public Health, the national body providing nursing programs. There are two programs of basic nursing education, one for a two-year certified nurse and one for a three-year state nursing diploma. Access to these two training programs is obtained by direct or professional competition. For the direct competition, the candidates for the diploma of certified nurse must have the general certification of secondary education, and candidates for the State diploma Nursing program must have the general certification of secondary education and 13 years of general education. The professional competition is open to registered nurses and state nurses working in the government who have the required five years of professional experience in their positions. Following the basic nursing education, there is training for executive nurses integrating multiple sectors (nursing, otorhinolaryngology, anesthesiology-resuscitation, surgery, ophthalmology, health and safety at work, mental health, epidemiology, and pediatrics), which will lead to their upgradation to “specialist nurses”. Access to this training is made by competition and concerns state-qualified nurses with a professional seniority of 3 to 5 years. The executive nurses intervene in a clinical environment (advanced practice), in training and in management. Finally, training for a master’s or doctoral degree in universities is not available at the moment in Burkina Faso, but rather takes place abroad.

### Organization of epilepsy care

The number of neurologists, neurosurgeons and psychiatrists is also limited. They provide epilepsy care only in four of the five tertiary hospitals and private medical clinics in Ouagadougou, Bobo Dioulasso and Ouahigouya. In other regions of the country, the epilepsy care is provided by general practitioners, specialist nurses in psychiatry, and nurses and midwives working in primary health. The National League Against Epilepsy and National Neurological Society has organized post-graduate training in neurology and continuing medical training in epilepsy for general practitioners, medical students and nurses since 2014. They also conducted public awareness campaigns during the Epilepsy Days. Concerning diagnosis, cerebral imaging is available in Ouagadougou and Bobo Dioulasso. Electroencephalogram is only available in Ouagadougou, in two private medical clinics and in one tertiary hospital. The price of electroencephalogram service was about 30.5 euro in Ouagadougou. The main drugs available, in generic forms, are phenobarbital, carbamazepine and diazepam. Other drugs (sodium valproate, lamotrigine, and levetiracetam) are also available in Ouagadougou and Bobo Dioulasso. Injectable forms of diazepam and phenobarbital are available for the treatment of status epilepticus.

### Study profile

This was a cross-sectional study conducted in Ouagadougou, the capital city of Burkina Faso, from August 1^st^ to September 15^th^, 2017. The city of Ouagadougou has a population of 3 million. The population investigated included nurses working in the health districts of Ouagadougou at the time of this study. The paramedical staff gave their consent to participate in the research study. Specialist nurses in psychiatry were not included in this study.

### Sampling

#### Size of the study population

For the calculation of the sample size, we used the following formula: n= *Z*^2^*P* (1-*P*) / *d*^2^, in which *Z* (écart type) = 1.96 for the 95% confidence level, *P* (proportions de la population d’étude) = 50%, *d* (precision desired) = 0.05. According to this formula, the number of nurses who must be included in the study (n) was 251.

#### Sampling procedure

We chose to survey 20% of health facilities in the city of Ouagadougou (*n* = 109). Three districts were randomly selected to carry out the survey (Kossodo, Baskuy and Signoghin). In each district, we performed a stratified sampling of health facilities according to the size of the health district, i.e., five health facilities for the small district and eight for the others. In the selected health facilities, we carried out a systematic sampling of the participants.

### Data collection

#### Data collection instrument

We developed a 25-item questionnaire containing information on demographics (6 items), personal experience with epilepsy (3 items), knowledge of epilepsy diagnosis (25 items), knowledge of epilepsy care (5 items), type of epilepsy care education received in the past (1 item), and perspective of how people with epilepsy or epilepsy is viewed by others (3 items). This questionnaire included socio-professional data of participants (age, sex, seniority in nursing practice, qualification, work place, and level of education), data on their knowledge of epilepsy (definitions of epilepsy, clinical manifestations, types of seizure, causes of epilepsy, triggering factors for epilepsy, treatment of epilepsy, medications, route of administration of antiseizure medication (ASM) for status epilepticus, duration of ASM therapy, and treatment of epilepsy) and attitudes of nurses toward a generalized epileptic seizure. Administration and data collection were performed by Fatimata Kinda.

#### Pretesting of the questionnaire

A pilot study was conducted at two CMAs, one dispensary and one maternity, in order to test if the tool could be easily completed and understood by each participant. The questionnaire prepared in the French language was pretested among 50 participants (10 midwives, 20 nurses and 20 registered nurses). Each participant was required to comment on whether there were any questions requiring improved clarification or change. Pre-test participants were not included in this study.

#### Data collection

After the approval of this study by the Regional Health Directorate of the Centre, we presented the objectives of the study to the officials of the selected health centers. Questionnaires were also self-administered in morning staff meeting after a brief introduction of the study and nurses who gave their consent completed the questionnaires. Depending on the availability of participants, the completed forms were either collected on-site or collected by the Head of Post Nurse and then sent to Fatimata Kinda. The survey lasted for 6 weeks.

#### Data analysis

Data were analyzed with the Chi-square test by using Epi Info version 7. *P* value less than or equal to 0.05 was considered as statistically significant.

### Assessments

#### Knowledge Score

Low knowledge was defined as a proportion of correctly answered knowledge-related questions < 50%, while good knowledge was defined as a proportion ≥ 50%.

#### Management Score

Low management was defined as a proportion of correctly answered practice-related questions regarding pediatric epilepsy < 50% while high management was defined as a proportion ≥ 50%.

## Results

### Socio-professional characteristics

Of the 251 participants preselected, 213 (84.9%) consented to participate, including 115 (54%) females and 98 (46%) males. The mean age was 39.5 years. The age group between 40–49 years was the most represented (46.6%). According to the education level, most of the respondents had a general certification in secondary education (170; 79.81%) and high school diploma (43; 20.19%). The average number of years of nursing practice was 13 years (range, 1 to 33 years). The majority of participants (62%) had a professional experience of more than 10 years. Concerning the participant qualification, 87 had a state diploma of nurse (40.8%), 60 were assistant nurses (28.2%) and 66 were midwives (31%). The majority of respondents were working in CSPS (76%) and the remaining (24%) in district hospitals. Table 1 highlights the social-professional characteristics of the participating nurses.

**Table 1:** Demographics of participants (*n* = 213)

	***n***	**Percentage (%)**
**Age group (years)**		
20–29	24	11.3
30–39	68	31.9
40–49	99	46.5
50–59	22	10.3
**Gender**		
Male	98	54
Female	115	46
**Workplace**		
Dispensary	162	76.1
District hospital	51	23.9
**Educational level**		
General certification of secondary education	170	41.3
High school diploma	43	15
**Nurse qualification**		
Licensed practical nurses	87	40.9
Certified nurses	60	28.1
Midwives	66	31
**Senior-level experience (years)**		
1–5	21	9.9
6–10	58	27.4
11–15	58	27.4
16–20	45	20.9
> 20	31	14.4

### Experience and familiarity with epilepsy

All the participants said that they had ever studied epilepsy in nursing school. Two hundred and eleven (99%) participants said they did not receive any training on epilepsy-related care during the last 6 months before the study. All participants were willing to receive either theoretical learning or clinical training on epilepsy. The main sources of epilepsy information were the relatives (51.2%), training school (39.9%), medical staff (19.7%) and media (17.8%). One hundred and sixty-three participants (76.4%) said they had already witnessed an epileptic seizure. Most of the participants had witnessed an epileptic seizure (59.6%) in a hospital ward. Most of the participants had already provided care to an epileptic patient (63.4%). Table 2 highlights the experience and familiarity of participants with epilepsy.

**Table 2:** Familiarity with epilepsy (*n* = 213)

	***n***	**Percentage (%)**
**Epilepsy information sources**		
Nursing school	85	38.5
Media	38	17.8
Medical sources	42	19.7
Relatives	109	51.2
**Receiving epilepsy training in the last 6 months**		
Yes	0	0
No	213	100
**Have you ever witnessed a seizure attack?**		
Yes	163	76.5
No	50	23.5
**Have you ever provided care to an epileptic patient?**		
Yes	135	63.4
No	78	36.6
**Where did you witness a seizure ?**		
At a medical center	97	59.5
At home	12	7.4
At street	54	33.1
**Do you think that epilepsy training is necessary for you?**		
Yes	213	100
No	0	0

### Knowledge of epilepsy

Concerning the diagnosis of epilepsy, 194 (91.3%) participants answered correctly that epilepsy is a disease with repetition of seizures while 19 (8.7%) considered that a seizure was sufficient for a diagnosis of epilepsy. Most participants (52.6%) knew the main causes of epilepsy. The most commonly considered causes were brain disease (53%), complications during pregnancy and problems of childbirth (21.1%). Seventy-four (34.7%) participants had knowledge of more commonly triggering factors of seizures, including emotion (50%), irregular use of drugs (18%), fever (15%), and sensory stimulations (1.1%). All the participants had knowledge of the main symptoms of epilepsy, such as convulsion (58.2%), hypersalivation (29.3%), episodes of loss of consciousness (15.2%) and urination (2.1%). For the knowledge of major types of seizures, all the participants (100%) had adequate knowledge of generalized seizures, but none of them recognized focal seizures. Most of the participants (69%) answered correctly that epilepsy is not a contagious disease. All the participants gave correct answers on the main complications of generalized seizures, which are injuries (94.8%) and death (12.7%). All the participants were capable of naming one antiepileptic drug (100%). Most of the participants (92.9%) had knowledge of ASM used for the treatment of status epilepticus, mentioning phenobarbital (53%) and diazepam (40%). Most of the participants had knowledge on the main route of administration of diazepam (98.1%) and phenobarbital (94%) in the management of status epilepticus. All the participants knew the complications of epilepsy if left untreated, including increased seizures (84.8%), death (13%) and cognitive impairment (2.2%). Eighty-three (39.1%) participants answered correctly that epilepsy is curable. Seventy-nine (37.1%) knew that an ASM should be discontinued 2 to 5 years after seizure freedom. Overall, 142 (66.7%) of the participants had a good knowledge on epilepsy (score ≥ 50%). Table 3 summarizes the answers of the participants to questions on epilepsy knowledge in the questionnaire. The factors influencing the knowledge on epilepsy were participants’ qualification (*P* = 0.00075) and level of education (*P* = 0.001) (Table 4).

**Table 3:** Frequency distribution of participants knowledge of epilepsy (*n* = 213)

Variable	*n*	Percentage (%)
**Define epilepsy?**		
Current seizures	194	91.3
One seizure	19	8 .7
**What are the causes of epilepsy?**		
Brain disease	**113**	**53.1**
Difficulties during pregnacy	45	8
Infection	42	19.7
Inexplained	39	18.3
Don’t known	31	14.5
**Triggering factors**		
Emotion	107	50
ASM discontinuation	38	18
Fever	32	15
Alcohol	2	1.1
Sensory stimuli	2	1.1
Don’t known	32	14.8
**What are the major types of seizures?**		
generalized seizures	213	100
focal seizures	0	0
**What are the signs of a seizure attack?**		
Convulsion	191	89.7
hypersalivation	62	29.1
Loss of conscience	32	15
Urination	5	2.3
**Is epilepsy a contagions disease?**		
Yes	23	10.8
No	147	69
Don’t known	43	20.2
**Complications of a generalized seizure**		
Self-hurt	202	94.8
Hurt to others	35	16.4
Death	27	12.7
**What are the drugs used in the treatment of epilepsy?**		
Phenobarbital	213	100
Diazepam	213	100
Carbamazepine	87	40.8
Sodium valproate	76	35.7
Lamotrigine	10	4.7
**Medications for treatment of status epilepticus**		
Phenobarbital	113	53
Diazepam	85	39.9
Primidone	15	7
**Route of administration of diazepam for status epilepticus**		
Rectal	200	94
Intramusculaire	9	3.8
Nasal	2	1.1
Intrathecale	2	1.1
**When to discontinue the AED treatment?**		
Seizure freedom	43	20.2
At least 2 years after the last seizure	39	18.3
At least 5 years after the last seizure	40	18.8
Don’t known	71	33.3
**Complications of epilepsy if untreated**		
Increased seizures	181	85
Death	27	12.7
Cognitive disorders	5	2.3
**Is epilepsy curable ?**		
Yes	84	39.1
No	74	34.8
Don’t known	55	26.1

**Table 4:** Factors associated with good epilepsy knowledge among nurses

**Variable**	Good (*n* = 142)	Poor (*n* = 71)	*P* value
**Qualification**			
Licensed practical nurses (*n* = 87)	54 (62.1%)	33 (37.9%)	0.000075
Certified nurses (*n* = 60)	43 (71.6%)	17 (28.4%)	
Midwives (*n* = 66)	45 (68.2%)	21 (31.8%)	
**Level of education**			
GCSE (*n* = 162)	110 (67.9%)	52 (32.1%)	0.001
HSD (*n* = 51)	32 (62.7%)	19 (37.3%)	
**Senior-level experience (years)**			
0–10 (*n* = 79)	46 (58.2%)	33 (41.8%)	0.18035
11–20 (*n* = 103)	74 (71.8%)	29 (28.2%)	
> 20 (*n* = 31)	22 (70.9%)	9 (29.1%)	
**Workplace**			
CSPS (*n* = 162)	120 (74.1%)	42 (25.9%)	> 0.05
CMA (*n* = 51)	22 (43.1%)	29 (56.9%)	

*GCSE* Generalized certification of secondary school, *HSD* High school diploma

### Practices toward epilepsy

For questions regarding the first aid for patients with generalized seizures, 87 (40.8%) participants answered correctly. Forty-eight (22.5%) participants knew it is necessary to put the patient in a safe position and 18.3% answered that they would observe seizure symptoms carefully. After the seizure, 60 (28.2%) participants would reassure the patient and 20.8% would refer the patients to a higher level of support. Overall, 65.2% (*n* = 139) of participants had a good ability to manage epilepsy. Table 5 highlights the practices of participants toward epilepsy. The factors influencing the practices of participants were their qualification (*P* = 0.00005) and their workplace (*P* = 0.001) (Table 6).

**Table 5:** Attitudes and practices of participants toward epilepsy

	***n***	**Percentage (%)**
What would you do for a patient experiencing a tonic–clonic seizure?		
To refer the patient	103	70.9
Put the patient in a safe position	48	22.500
Hold the person and try to constrain	18	8.5
Observe seizure symptoms carefully	39	18.3
Don’t known	5	2.3
What should be done after a seizure?		
Reassure the patient	60	28.2
Give an injectable ASM	68	31
Refer the patient to higher-level support	44	20.6
Don’t known	41	19.4

**Table 6:** Factors influencing good practices toward epilepsy

Attitudes toward epilepsy			
	Good (*n* = 139)	Poor (*n* = 74)	*P* value
**Qualification**			
Licensed practical nurses (*n* = 87)	58 (66.7%)	29 (33.3%)	0.0005
Certified nurses (*n* = 60)	38 (63.3%)	22 (36.7%)	
Midwives (*n* = 67)	43 (64.2%)	23 (35.8%)	
**Senior-level experience (years)**			
0–10 (*n* = 79)	49 (62%)	30 (38%)	0.1805
11–20 (*n* = 103)	68 (66%)	35 (34%)	
> 20 (*n* = 31)	22 (70.9%)	9 (29.1%)	
**Workplace**			
CSPS (*n* = 162)	100 (61.7%)	62 (38.3%)	0.0001
CMA (*n* = 51)	39 (76.5%)	12 (23.5%)	

*GCSE* Generalized certification of secondary school, *HSD* High school diploma

## Discussion

The objective of this study was to determine the level of knowledge and attitude toward epilepsy among nurses and midwives working in health districts in Burkina Faso.

### Level of knowledge on epilepsy

In this study, most of the participants had knowledge on the causes of epilepsy. Brain disease was the most mentioned cause of epilepsy by the participants. These findings were similar to those of several studies in Brazil [11], Japan [12], Bhuta Kingdom [13] and Palestine [14]. Birth defects were cited as another cause of epilepsy by 21.12% of the participants, a percentage lower than in the study by Nishina et al. in Japan (46.7%) [12]. This difference could be explained by the lack of specific training of health workers on epilepsy in Burkina Faso. Infectious diseases were mentioned by 19.7% of the participants as a possible cause of epilepsy, a percentage also lower than in the study by Nishina et al. in Japan (27.0%) [12] and in the study by Harimanana et al. in Laos (38.3%) [15]. Moreover, 31% of the participants considered that epilepsy was a contagious disease, similar to the percentage in the study in Laos (33.3%) [15]. Almost all nurses in Palestine considered that epilepsy was a contagious disease (97%) [14]. These considerations may be related to the high frequency of febrile seizures in children. In this study, the symptoms of epilepsy were well known by the participants, in line with the study by Nishina et al. in Japan [12]. The most common symptoms recognized by the participants were convulsions (58.2%), hypersalivation (29.3%) and loss of consciousness (15.2%). However, convulsion and loss of consciousness were less known compared to the study by Nishina et al. in Japan [12], which reported that 90.9% and 90.2% of home nurses recognized convulsion and loss of consciousness as symptoms of epilepsy. In addition, 34.7% of participants were aware of the triggering factors of seizures (fever, alcohol consumption, sensory stimulation, and discontinuation of ASM). ASM discontinuation in patients with epilepsy was less recognized in our study (18%), compared to the study by Nishina et al. in Japan (89.5%) [12]. Moreover, 50% of participants identified emotion as a triggering factor of seizure. This could mean possible confusion with psychogenic attacks, which are more common in adolescents. All the participants were aware of the complications of generalized seizures. Injuries (97.3%) and death (2.7%) were the most considered complications of generalized seizure. As in our study, death was also rarely cited by the nurses in Laos (6.3%) [15]. Almost all participants were aware of status epilepticus medications (93%) and their route of administration (93%). Phenobarbital and diazepam are the only ASMs available in tertiary or in peripheral health centers in Burkina Faso [16]. All the participants knew at least one drug used for the treatment of epilepsy. However, Harimanana et al. reported a much lower percentage in Laos (59%) [15]. This difference could be explained by the availability of ASMs in these two countries. Regarding the duration of antiepileptic treatment, only 18.2% of participants knew that an ASM should be discontinued 2 years after seizure freedom, and the percentage was even lower in the study of Harimanana et al. [15]. In addition, 35% of the participants considered epilepsy as an incurable disease, while the majority of nurses in Turkey (74.8%) [17] and almost all nurses in Palestine (92.8%) [14] believe that epilepsy is incurable. This difference could be explained by the beliefs and experiences of the participants. Complications related to untreated epilepsy were widely known among the participants. Increase of seizures was the main complication reported by the participants (84.8%), comparable to that reported by Brizzi et al. (76.0%) [13]. On the other hand, death as a complication was better known among the nurses in the study by Brizzi et al. (13% versus 66.7%) [13]. Despite the low percentage (2.2%) of participants, cognitive disorders are important complications among epileptic patients.

### Level of practices toward epilepsy

In this study, 40.7% of participants had a positive attitude toward first aid for an epileptic patient experiencing generalized tonic clonic seizures; 18.3% would observe the seizure symptoms carefully and 22.5% would take measures to prevent injury. In the study by Nishina et al. in Japan, the majority of nurses said they would prevent injury (90.9%), prevent choking (87.7%) and observe seizure symptoms carefully (84.2%) [12]. Furthermore, 70.9% of the participants in this study said they would refer the patient to a higher-level health center. This indicates an insufficiency in the care of patients and the fear of being infected by the patient. In addition, most of the participants had a positive attitude (48.8%) at the end of the seizure, reassuring the patient (28.2%) and referring the patient to a higher-level support (20.6%). However, some participants (31%) said that they would prevent the onset of seizures in others by injecting them with an ASM; this could be fatal and result in a high risk of coma and death.

### Factors influencing the level of knowledge and practice

A high percentage of participants (66.5%) had a good level of knowledge on epilepsy and 65% had positive attitudes toward epilepsy. This level of performance could be explained by their high educational level, their professional experience, and their familiarity with epilepsy. Regarding educational level, more than 20% of them had a higher educational level than required for their qualification. The work experience was on average 13 years and 62.9% of them had more than 10 years of seniority. This professional experience is significant in our context due to the high mobility of staff. In a study in Japan, the nurses had a higher professional experience of 22.6 ± 9. 8 years [12]. The participants in our study were highly familiar with epilepsy. They had ever learned epilepsy-related knowledge in nursing school, had already witnessed an epileptic seizure (76.4%) and had provided care for an epileptic patient (63.4%). These proportions of having classes during initial training and witnessing seizures were similar to that in the study by Nishina et al. (60.7%) [12]. The majority of participants in our study were informed by medical sources, namely, during their initial training (39.9%) and from medical staff (19.7%). The incorrect answers to questions in the survey might be linked to the fact that almost all the participants had not received training on epilepsy-related care in the last six months. This justifies training of the participants in the management of epilepsy. Indeed, several authors had demonstrated that the level of nurses' knowledge can be improved by continued educational nursing programs [6, 18, 19]. Continuing medical education in epilepsy for nurses is available in our context, but there are no graduation courses. It would be interesting for the National League Against Epilepsy to offer an education program for paramedical personnel, as is done in Europe with epilepsy nurse specialists. Our study also revealed a significant link between the level of knowledge and the educational level, in agreement with several studies in Zambia [4] and Turkey [20]. Meda et al. in Burkina Faso [10] reported that the factors influencing the knowledge of health personnel are the duration as a health worker, experience of taking care of an epileptic-seizure patient, having previously done public awareness activities on epilepsy, and having already prescribed anti-epileptics drugs. Our study also highlighted for the first time a link between the level of knowledge and the qualification of participants. In this study, the registered nurses had higher levels of knowledge than state nurses and state midwives. This can be explained by the fact that the registered nurses mainly have caring tasks while state nurses also have supervision activities in addition to care tasks. As for state midwives, their duties are also to provide care. Our study found a link between the level of practice and the place of work. Participants working in district hospitals had more practice than those working in dispensaries. It should be noted that most participants worked in district hospitals (59.5%) with general practitioners and specialist physicians who theoretically have a higher level of knowledge and practice on epilepsy.

### Limitations of the study

This cross-sectional study had several limitations. First, the initially expected number of participants could not be reached, leading to a decrease in the power of the results. Indeed, due to the high workload of participants and the short duration of data collection, many participants were not able to be enrolled in the study. In addition, we noted the reluctance of some participants to take part in the investigation. Second, the fact that the questionnaire was self-administered could lead to a bias in the interpretation of the results. Third, the threshold for assessing nurses' appropriate level of knowledge and practice was relatively low, which could lead to an overestimation of nurses' skill levels.

## Conclusions

The results of this survey indicate that the participants working in health districts of Ouagadougou (Burkina Faso) have a good level of knowledge of epilepsy and management strategies for epilepsy. The knowledge and attitude of participants depend on their qualification and educational level. It is important to provide continuing education for nurses to improve their abilities to deal with an epileptic seizure event.

## Data Availability

The data are available from the corresponding author.

## Abbreviations

ASM: Antiseizure medication
CHR: Regional referral hospital
CMA: District referral hospitals
CSPS: Health and Social Promotion Centers
